# Delivery of magnetic resonance-guided single-fraction stereotactic lung radiotherapy

**DOI:** 10.1016/j.phro.2020.05.002

**Published:** 2020-05-20

**Authors:** Tobias Finazzi, John R. van Sörnsen de Koste, Miguel A. Palacios, Femke O.B. Spoelstra, Berend J. Slotman, Cornelis J.A. Haasbeek, Suresh Senan

**Affiliations:** Department of Radiation Oncology, Amsterdam University Medical Centers, Location VUmc, de Boelelaan 1117, 1081 HV Amsterdam, The Netherlands

**Keywords:** SABR, Single-fraction, MR-guided radiation therapy, Adaptive radiotherapy, Lung cancer

## Abstract

**Background and purpose:**

Single-fraction stereotactic ablative radiotherapy (SABR) is an effective treatment for early-stage lung cancer, but concerns remain about the accurate delivery of SABR in a single session. We evaluated the delivery of single-fraction lung SABR using magnetic resonance (MR)-guidance.

**Materials and methods:**

An MR-simulation was performed in 17 patients, seven of whom were found to be unsuitable, largely due to unreliable tracking of small tumors. Ten patients underwent single-fraction SABR to 34 Gy on a 0.35 T MR-linac system, with online plan adaptation. Gated breath-hold SABR was delivered using a planning target volume (PTV) margin of 5 mm, and a 3 mm gating window. Continuous MR-tracking of the gross tumor volume (GTV_t_) was performed in sagittal plane, with visual patient feedback provided using an in-room monitor. The real-time MR images were analyzed to determine precision and efficiency of gated delivery.

**Results:**

All but one patient completed treatment in a single session. The median total in-room procedure was 120 min, with a median SABR delivery session of 39 min. Review of 7.4 h of cine-MR imaging revealed a mean GTV_t_ coverage by the PTV during beam-on of 99.6%. Breath-hold patterns were variable, resulting in a mean duty cycle efficiency of 51%, but GTV_t_ coverage was not influenced due to real-time MR-guidance. On-table adaptation improved PTV coverage, but had limited impact on GTV doses.

**Conclusions:**

Single-fraction gated SABR of lung tumors can be performed with high precision using MR-guidance. However, improvements are needed to ensure MR-tracking of small tumors, and to reduce treatment times.

## Highlights

• MR-guidance enables high precision single-fraction lung SABR delivery.
• Breath-hold gating resulted in a mean tracked GTV_t_ coverage of 99.6% during beam-on.
• On-table plan adaptation improved PTV coverage, but had little impact on GTV doses.
• Improved techniques are needed to allow for consistent MR-tracking of small tumors.

## 1 Introduction

Stereotactic ablative radiotherapy (SABR) is the guideline-recommended treatment for medically inoperable early-stage non-small cell lung cancer (NSCLC) [1], [2]. SABR can also improve survival in patients with oligometastatic disease [3]. Various dose fractionation schedules have been reported, and a biologically effective dose (BED_10Gy_) ≥100 Gy has been recommended for primary lung tumors [4].

Delivery of SABR in a single fraction is a potentially more convenient approach for patients, and the safety and efficacy of single-fraction SABR has been demonstrated for both early-stage NSCLC and pulmonary metastases [5], [6], [7], [8], [9]. However, clinical use of single-fraction SABR does not appear to be widespread, in part due to concerns about the accuracy of SABR delivery. One approach to improve accuracy is by using internal fiducial markers as a surrogate for x-ray based gating or tumor tracking [10]. However, the implantation of fiducials is not without risks, especially in the elderly and frail patients [10], [11], [12]. Approaches for tracking lung tumors without using fiducials have also been developed, but their reliability depends on tumor size and density [13], [14]. Fast delivery of single-fraction lung SABR can be performed using flattening-filter-free (FFF) volumetric modulated arc therapy (VMAT), using an internal target volume (ITV) approach [15]. However, active motion monitoring is desirable as both 4-dimensional (4D) computed tomography (CT) and cone-beam CT (CBCT) may underestimate tumor motion during lung SABR [16].

Magnetic resonance (MR-)guided radiotherapy may facilitate single-fraction treatments as it permits SABR delivery under continuous image guidance [17]. Real-time MR-guidance circumvents the need for implanted markers, and allows for a more accurate assessment of respiratory-induced tumor motion when compared to use of a pre-treatment 4DCT [18]. In addition, use of gated delivery and daily on-table plan adaptation can allow for both optimization of target coverage and reduction in organ at risk (OAR) doses [19], [20], [21], [22]. We report on our early experience with treating lung tumors in a single fraction, using the so-called stereotactic MR-guided adaptive radiation therapy (SMART) approach.

## 2 Materials and methods

### 2.1 Introduction of single-fraction SMART

Single-fraction SABR of lung tumors has been an option in our departmental protocol since the safety and efficacy of this approach was reported in a prospective study [23]. Since late 2018, suitable patients with lung tumors were evaluated for single-fraction SMART on the MRIdian MR-linac (ViewRay Inc., USA). Patients were eligible if they fulfilled eligibility criteria used in the Radiation Therapy Oncology Group (RTOG) 0915 study, namely a tumor located ≥2 cm from the proximal bronchial tree and measuring ≤5 cm [23]. In addition, SMART was considered when delivery was technically challenging, for example if tumors were mobile and/or when clinicians were concerned about single-fraction delivery when using an ITV approach. This retrospective analysis was approved by the institutional ethics committee.

Treatment simulation and delivery were performed on the MR-linac, which has been in use in our institution since April 2018. The MR-linac incorporates a 0.35 T MR scanner and a linear accelerator delivering 6 MV FFF photons at a dose rate of 630 MU/min. The dose rate of our previous MRIdian Cobalt-60 system was considered unsuitable for single-fraction lung SABR due to long treatment times. The simulation and delivery procedures have been described previously [19]. Briefly, a 3-dimensional (3D) MR scan was first acquired during a 17-s breath-hold. Subsequently, tumor motion was sequentially observed in all 3 planes using MR cine imaging with audio coaching, during normal respiration and in both quiet inspiratory and expiratory breath-holds. The patterns of tumor motion and position were observed visually in order to identify an optimal phase for gated delivery. The phase chosen depended on tumor visibility, distance to the chest wall, as well as breath-hold reproducibility and tolerance, with most patients finally treated in shallow inspiration. Finally, real-time tumor tracking was evaluated in a sagittal MR plane which was generally in the middle of the tumor volume, using a slice thickness of 5 mm, but occasionally 7 mm. Tracking of a sagittal tumor outline was performed using the proprietary deformable image registration software. Briefly, the system acquired a series of preview MR cine images, from which it selected a reference (key) frame that best matched the sagittal 3DMR plane chosen for tracking. The tracking algorithm then automatically deformed the gating contour from the key frame to each acquired MR cine image at 4 frames per second [24]. Tracking performance was then assessed visually by a clinician and physicist present at the console.

After MR-simulation, a breath-hold planning CT scan was acquired for purposes of dose calculation, and for verifying tumor size and shape. After rigid co-registration of the CT to the planning 3DMR scan, the gross tumor volume (GTV) was contoured by a clinician on the breath-hold CT scan, before the same clinician contoured the GTV on the corresponding breath-hold 3DMR scan. Any deviations in volume or shape observed between GTV contours on CT versus MR were reviewed by a second clinician, and a consensus was reached. Following delineation of the GTV and OARs on the 3DMR scan, a planning target volume (PTV) was created by adding an isotropic margin of 5 mm to the GTV. A step-and-shoot intensity modulated radiotherapy (IMRT) plan was then created in the MRIdian system, using a Monte Carlo algorithm with a dose calculation grid size of 2 mm, and 1% statistical uncertainty. Electron density maps were derived from planning CT scans, which were deformably registered to the respective 3DMR scans during offline and on-table adaptive planning. The accuracy of this deformable image registration, which accounted for potential differences in breath-holds between CT and MR images, was assessed by the radiation therapist and/or physicist. The magnetic field was taken into account for both the fluence optimization and final dose calculation of all plans [25], [26], [27].

On the day of treatment, a new breath-hold 3DMR scan was acquired in treatment position, using the same respiratory instructions as used for simulation. After rigid fusion to the GTV on the baseline MR, OAR contours were deformably propagated to the MR-of-the-day, and edited as needed. GTV contours were modified by the clinician present only if this was considered necessary after visual assessment. The baseline plan was recalculated on the anatomy of the day, the so-called «predicted» plan. Hereafter, the IMRT plan was reoptimized based on the (adapted) GTV and OARs, using the same beam setup and optimization objectives as in offline planning. The planning objective was to deliver a prescription dose (PD) of 34 Gy to 95% of the PTV (V_34Gy_ ≥ 95%; V_47.6Gy_ ≤ 1 cm^3^), while maintaining compliance with OAR constraints used in the RTOG 0915 study [23]. Clinicians then selected either the on-table reoptimized plan, or the baseline plan for delivery [19], [26].

On-table plan quality assurance (QA) was performed using an independent Monte Carlo dose calculation engine available with the MRIdian online adaptive workflow. Treatment delivery was performed during breath-holds, with continuous visualization of the tracked GTV (GTV_t_) in a sagittal MR plane, acquired at 4 frames per second. The beam was automatically turned off when a pre-specified maximum proportion of the GTV_t_, the so-called threshold-region of interest percentage (ROI%), was outside the gating window boundary. The gating window boundary was created by adding an isotropic margin of 3 mm to the breath-hold GTV. To facilitate patient breath-holds, both the GTV_t_ and the gating window boundary were projected to the patient on an in-room monitor in real-time (Supplementary Fig. 1). Due to lengthy delivery times, treatment plans were divided into two equal parts delivering 17 Gy each, and a breath-hold 3DMR scan was repeated mid-treatment, with the option for plan re-adaptation. This approach also allowed for a short mid-treatment break should the patient require it.

### 2.2 Patients

Between October 2018 and November 2019, 17 patients were evaluated using MR simulation for single-fraction SMART, and 10 were identified as being suitable for treatment. Seven patients were considered unsuitable for MR-SABR for reasons including suboptimal GTV tracking due to adjacent blood vessels (n = 4), and limited visibility of a sub-centimeter tumor (n = 1). The average tumor diameter on CT images for these five simulation failures was 1.1 cm (range, 0.9–1.2 cm). Other reasons for deciding against single-fraction SMART were the proximity to chest wall (n = 1) and a patient with severe chronic obstructive pulmonary disease who was unable to perform repeated breath-holds. Of the MR-simulation failures, five patients subsequently underwent 1- or 3-fraction SABR delivered using an ITV-based approach on a conventional linear accelerator. Another patient received 3 fractions of 18 Gy on the MR-linac, and a wait-and-see approach was chosen for a patient with a small lung metastasis.

**Fig. 1:**
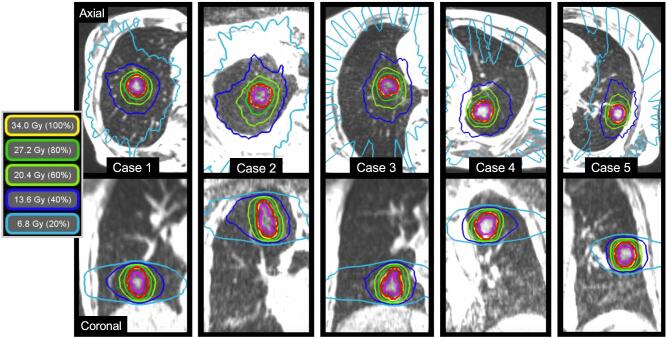
Breath-hold 3-dimensional (3D) magnetic resonance (MR) images of the first five patients treated with single-fraction lung stereotactic ablative radiotherapy using MR-guidance. The 3DMR scan is acquired on the MR-linac during a 17-second breath-hold, using a TrueFISP sequence with 1.6 mm × 1.6 mm × 3.0 mm resolution. Using the stereotactic MR-guided adaptive radiation therapy approach, one fraction of 34 Gy is delivered to the planning target volume (red), which is created by adding a 5 mm isotropic margin to the breath-hold gross tumor volume (purple). (For interpretation of the references to color in this figure legend, the reader is referred to the web version of this article.)

The characteristics of 10 patients scheduled to undergo single-fraction SMART are summarized in Supplementary Table 1. A decision to perform SABR without histological confirmation was taken only following multidisciplinary discussion, and in accordance with clinical practice guidelines [1]. Median patient age was 73 years (range, 58–80 years), and the median Eastern Cooperative Oncology Group Performance Status (ECOG PS) was 1 (0–2). Indications for SABR were a biopsy-proven NSCLC (n = 1), clinically diagnosed lung cancer (n = 7), or lung metastasis (n = 2). Median GTV and PTV at baseline were 2.9 cm^3^ (range, 1.8–6.5 cm^3^) and 10.1 cm^3^ (7.5–20.5 cm^3^), respectively. PTV coverage by the PD (V_34Gy_) was 95.0% in all baseline plans, equating to delivery of a BED_10Gy_ of 149.6 Gy to 95% of the PTV. The maximum dose, as percentage of PD, was a median of 138.3% (126.5–149.6%) within the GTV. Treatment plans of the first five patients are shown in Fig. 1.

### 2.3 Image and outcome analysis of single-fraction SMART

The stored real-time MR cine images depicting the GTV_t_ and gating window boundary in sagittal plane, were analyzed for each patient as described previously [17]. Briefly, the raw images were analyzed using ImageJ (v1.51i; National Institutes of Health, USA). In ImageJ, color thresholding was used to extract the areas encompassed by the GTV_t_, the gating window boundary, and both. The gating window boundary was isotropically expanded by 2 mm to recreate the PTV and measure the fraction of GTV_t_ inside the PTV during beam-on (GTV_t_ coverage). Centroid GTV_t_ positions were used for motion analysis. All data were saved in comma-separated values (CSV) file format and analyzed using MS Excel 2013 to estimate the GTV_t_ coverage, breath-hold patterns, and duty cycle efficiency. The latter was defined by the percentage of effective gating treatment time, namely the “beam-on” frames divided by the total number of MR cine frames acquired during treatment, including frames acquired during gantry rotation and multileaf collimator (MLC) motion.

Patients were followed for clinical outcomes, and clinical and imaging data were obtained from external institutions, when necessary. Toxicities were scored by at least two radiation oncologists, and graded using the Common Terminology Criteria for Adverse Events (CTCAE) version 5.0 [28].

## 3 Results

### 3.1 Treatment characteristics

Duration of a full single-fraction SMART session, as measured from the patient entering the changing room to the end of delivery, was a median of 120 min (range, 74–185 min). Nine patients completed treatment as scheduled, and reported no discomfort other than fatigue and mild musculoskeletal complaints immediately after completion of breath-hold SABR. The tenth patient developed back pain during a lengthy treatment session, and after receiving a dose of 25 Gy, completed the treatment on a subsequent day.

On the day of treatment, only minimal re-contouring of the GTV was deemed necessary by clinicians. The average GTV variation versus baseline was +0.2 cm^3^ (range, 0.0–0.8 cm^3^), or 6.4% (0.0–16.7%). Clinicians selected the on-table reoptimized plan-of-the-day for delivery in all but one patient, in whom PTV coverage was slightly higher than prescribed with the baseline (or predicted) plan. Overall, on-table plan adaptation improved PTV coverage by the PD (V_34Gy_) from an average of 89.8% in predicted plans, to 95.0% in reoptimized ones. This corresponded to increases in the biologically effective doses (BED_10Gy_) delivered to 95% of the PTV (D_95%_) from an average of 142.7 Gy (range, 135.1–153.6 Gy) in predicted plans, to 149.6 Gy in all reoptimized ones. Doses delivered to the GTV were similar, with an average GTV D_50%_ (median dose; BED_10Gy_) of 223.5 Gy (193.8–248.0 Gy) and 224.7 Gy (195.6–244.3 Gy), respectively, in predicted and reoptimized plans.

On mid-treatment 3DMR scans, treatment plans were again reoptimized in seven patients, even though improvements in target coverage were minimal (data not shown). A minor chest wall (V_22Gy_) violation was observed in one predicted and reoptimized plan each, both during mid-treatment plan adaptation, but both were deemed acceptable by clinicians [29]. In another patient, the mid-treatment plan adaptation avoided a hot spot in the chest wall (predicted vs. reoptimized: chest wall Dmax 38.1 vs. 34.0 Gy; V_22Gy_ 3.6 vs. 2.1 cm^3^). No other OAR violations were observed in any predicted or reoptimized plans.

### 3.2 Verification of single-fraction SABR delivery

A total of 7.4 h of MR cine imaging (105,951 frames) acquired during single-fraction SABR were analyzed (Table 1). SABR was delivered using an initial threshold-ROI% of 10% in all cases. In order to improve the duty cycle efficiency, the threshold-ROI% was increased during delivery to 15% in four patients, and to 20% in one patient. For the latter, visual assessment of the adequacy of tumor coverage was assessed on MR images for the revised thresholds. The GTV_t_ area encompassed by the 3 mm gating window during beam-on averaged 95.4% (5th–95th percentile, 88.1–100.0%) for the 10 patients. The maximum proportion of the GTV_t_ outside the gating window during beam-on did not exceed 0.1% of the preset threshold-ROI%.

**Table 1:** Details of magnetic resonance (MR)-guided single-fraction lung stereotactic ablative radiotherapy (SABR) delivery during repeated patient breath-holds. The beam is automatically turned off when a pre-specified proportion (so-called threshold-region of interest percentage; ROI%) of the tracked gross tumor volume (GTV_t_) is outside the 3 mm gating window boundary. Although the breathing patterns (Fig. 2) and resulting duty cycle efficiency were variable, excellent GTV_t_ coverage during beam-on was observed in all cases, using a planning target volume (PTV) margin of 5 mm. The SABR delivery session is the period during which patients are instructed to perform breath-holds, whereas the full stereotactic MR-guided adaptive radiation therapy (SMART) session reflects the entire in-room workflow, measured from the patient entering the changing room to the end of treatment delivery.

Case	Threshold-ROI%	Mean GTV_t_ coverage by the PTV during beam-on (5th–95th percentile)	Duty cycle efficiency	SABR delivery session (min)	Full SMART session (min)
1	10%–15%	99.0% (97.0–100.0%)	54%	38	74
2	10%	100.0% (100.0–100.0%)	85%	28	82
3	10%–15%	99.6% (98.7–100.0%)	34%	59	150
4	10%	99.9% (99.8–100.0%)	72%	34	86
5	10%–15%	99.3% (97.7–100.0%)	58%	36	102
6	10%–20%	99.2% (94.1–100.0%)	37%	57	143
7	10%	99.9% (99.6–100.0%)	52%	48	119
8	10%–15%	99.6% (97.9–100.0%)	56%	39	129
9	10%	99.9% (99.4–100.0%)	60%	37	120
10*	10%	99.9% (99.2–100.0%)	40%	66	185*

* Case 10 required SMART delivery in two sessions due to patient discomfort, and the total duration of both sessions is reported.

**Fig. 2:**
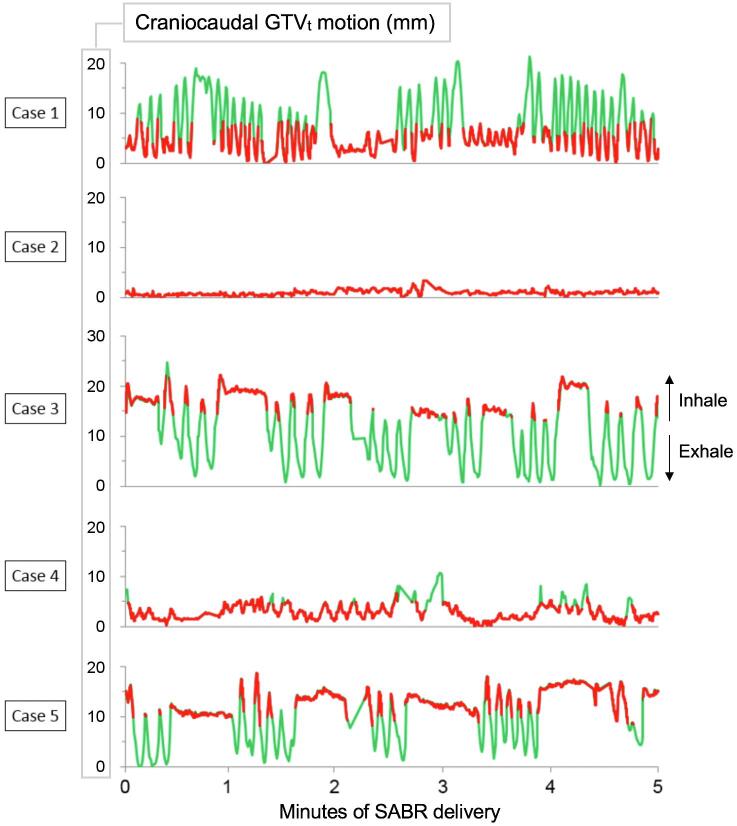
Breathing-induced craniocaudal tracked gross tumor volume (GTV_t_) motion on magnetic resonance (MR) imaging observed during single-fraction lung stereotactic ablative radiotherapy (SABR) for the first five patients. Beam-on is indicated by the red tracing when the GTV_t_ is in the correct position. The green tracing indicates beam-off when a prespecified fraction of GTV_t_ is outside the gating window boundary. One patient (case 2) produced a shallow curve due to limited tumor mobility, resulting in a high duty cycle efficiency. (For interpretation of the references to color in this figure legend, the reader is referred to the web version of this article.)

With use of a 5 mm PTV margin, the mean GTV_t_ coverage by the PTV during beam-on averaged 99.6% (5th–95th percentile, 98.0–100.0%) for all patients. We observed variability in breathing-induced tumor motion, shown in Fig. 2 for the first five patients, but this did not affect GTV_t_ coverage. Varying breath-hold patterns also resulted in variable duty cycle efficiency, which averaged 51% (range, 34–85%) for all patients. The median treatment delivery duration was 39 min (28–66 min), and this included beam-off phases between breath-holds, as well as gantry rotation and MLC motion. Real-time MR images acquired during treatment of the first five patients are available as Supplementary Video 1.

### 3.3 Early clinical outcomes

Nine of the 10 patients treated were alive at the time of this report. One death occurred in a 75-year old patient with a history of cardiovascular disease, who developed a fatal myocardial infarction 11 months following SABR to a peripheral lower lobe tumor (case 1). At a median follow-up of 5 months (range, 2–12 months), CTCAE grade ≥2 toxicities were as follows: one patient developed mild worsening of preexistant exertional dyspnea 10 weeks following SMART, consistent with symptomatic radiation pneumonitis (CTCAE grade 2) on CT imaging. This patient did not require medical treatment. Another patient reported persistent fatigue (CTCAE grade 2) for a few weeks after SABR, with spontaneous recovery. No CTCAE grade 3–5 toxicities, and no local recurrences, have been observed.

## 4 Discussion

MR-guided single-fraction lung SABR delivered during repeated breath-holds was generally well tolerated by patients. SABR was delivered with a high level of precision, as the average beam-on GTV_t_ coverage by the PTV in sagittal plane was 99.6%. However, some small tumors (average diameter 1.1 cm) were found to be unsuitable for MR-based tracking using the software available at that time.

To the best of our knowledge, this is the first reported experience of single-fraction lung SABR using an MR-assisted approach. We had initial concerns about the feasibility of single-fraction breath-hold lung SABR on the MR-linac due to the long delivery times, as well as technical challenges such as the stability of patient positioning, and the ability to treat small tumors eligible for single-fraction SABR. Our MR-guided approach is generally more complex, requiring longer delivery times than with FFF-VMAT [15], [19], [30]. Single-fraction SMART also involved additional mid-treatment simulation and plan assessment. In addition, the overall treatment time included discussions between members of the treatment team, all of whom needed to gain familiarity with the procedure. Longer on-table times may be acceptable when considering the resources spared with single-fraction treatments, and this may facilitate the scheduling of multiple SABR treatments between cycles of systemic therapy in oligometastatic patients [31]. However, further reductions in treatment times are needed in order to improve patient tolerance. We observed a variability in breath-hold patterns exhibited by patients, resulting in an average duty cycle efficiency of only 51%, although GTV_t_ coverage was not impaired with use of real-time MR guidance. Furthermore, only approximately 60% of patients who were assessed for this procedure ultimately underwent single-fraction SABR on the MR-linac, indicating that improved imaging and tracking software are required in order to allow for the treatment of small tumors in the range of 1 cm.

We acknowledge that respiratory gating, or tracking, can also be performed using both internal and external markers [32], [33], [34], [35], or with template matching and triangulation of kV images for markerless breath-hold lung SABR [13]. Both 4DCT and 4D cone-beam CT under-predict lung tumor motion during radiotherapy [16], and variations such as baseline drifts and shifts suggest that an active approach including real-time monitoring may be preferred when treating mobile tumors [18]. The demands for positional accuracy may be particularly high in single-fraction lung SABR, where inaccuracies are not mitigated by delivery in multiple fractions. There is a role for real-time image guidance and adaptive planning [36], with video-assisted MR-guidance being an attractive solution as it is without need for implanted fiducials, external surrogates, or additional radiation exposure [17]. Additional studies will be needed, however, to precisely quantify the accuracy of real-time MR-tracking of lung tumors [37], [38]. We continuously assessed tracking performance visually as the tracking algorithm could be compromised by image noise and artifacts. Furthermore, the real-time monitoring was only performed in one sagittal plane, leading to a risk of undetected lateral movement, which may be suspected when the tracked tumor area appears to decrease, or when the system indicates a low correlation of the tracking algorithm. In such situations, an additional 3DMR scan was performed for intra-fractional positional verification. In addition, we applied a PTV margin that is larger than the boundary used for MR-gating, in order to account for the remaining positional uncertainties. Improvements in gating precision are desirable as this may increase confidence to reduce PTVs. In peripheral lung tumors, MR-guided breath-hold SABR was shown to result in PTVs measuring only 54% of those required with an ITV approach [20]. Reducing lung irradiation is important as indications for repeating SABR are becoming more common for patients with both metastases and primary lung cancers [39], [40].

Our analysis suggests that on-table plan adaptation can improve PTV coverage, although the impact on GTV dose did not appear to be clinically relevant. Similar findings were observed for fractionated MR-guided SABR delivery for peripheral lung tumors [20]. Given the need for optimal techniques for single-fraction SABR, we continue to perform on-table plan adaptation as the additional workload of re-contouring is limited with respect to the total duration of each session. However, future studies may reduce the mid-treatment procedures employed in our initial 10 patients. In addition, new clinical software for tumor tracking at 8 frames per second and with different deformable registration software options is now undergoing evaluation, and may improve system performance. Due to uncertainties in MR-based contouring of some lung tumors, we will continue to use the breath-hold planning CT scan in order to verify tumor size and shape. Future studies are needed to address additional challenges such as susceptibility and motion artefacts in the thorax [41], [42].

Based on recent studies, single-fraction SABR is now a standard of care for medically inoperable patients with a peripheral stage I NSCLC. However, as local failure rates of 10% or higher have been reported after SABR for peripheral early-stage NSCLC [5], [43], [44], improvements in the delivery of radiotherapy remain desirable. The RTOG 0915 study demonstrated similar efficacy and toxicity of SABR delivered with 34 Gy in a single fraction, compared to 48 Gy in 4 fractions [5]. Similarly, no differences in tumor control or toxicity were seen for patients with medically inoperable stage I NSCLC in a study comparing 30 Gy in a single fraction versus 60 Gy in 3 fractions. The latter study also suggested that quality of life (QoL) measures of social functioning and dyspnea were better in the single-fraction arm, although QoL analyses were of exploratory nature [9]. Additional data will be forthcoming from a completed randomized phase II trial that evaluated single-fraction SABR for oligometastatic patients with 1–3 lung metastases, both in terms of clinical efficacy, as well as resource use and costs compared to SABR in 4 fractions [45].

In conclusion, single-fraction lung SABR using MR-guidance is feasible, and it allows for high-precision delivery. Improved imaging is needed to ensure tumor tracking in all patients who may be eligible for this approach, and faster workflows are needed to improve patient comfort and resource utilization.

## Declaration of Competing Interest

The authors declare the following financial interests/personal relationships which may be considered as potential competing interests: The Department of Radiation Oncology at the VU University Medical Center has funded research agreements with ViewRay Inc. and Varian Medical Systems. M.A.P. reports personal fees from ViewRay, Inc., outside the submitted work. C.J.A.H. and S.S. report personal fees from Varian Medical Systems, outside the submitted work. B.J.S. reports personal fees from ViewRay Inc. and Varian Medical Systems, outside the submitted work. T.F., J.R.S. and F.O.B.S. have nothing to disclose.
