# Supplementary material for: Delivery of magnetic resonance-guided single-fraction stereotactic lung radiotherapy
Source: Phys Imaging Radiat Oncol. 2020 May 20;14:17–23. doi: 10.1016/j.phro.2020.05.002 (PMC7807654; doi:10.1016/j.phro.2020.05.002)
Supplement: Supplementary Data 1 [file mmc1.docx]

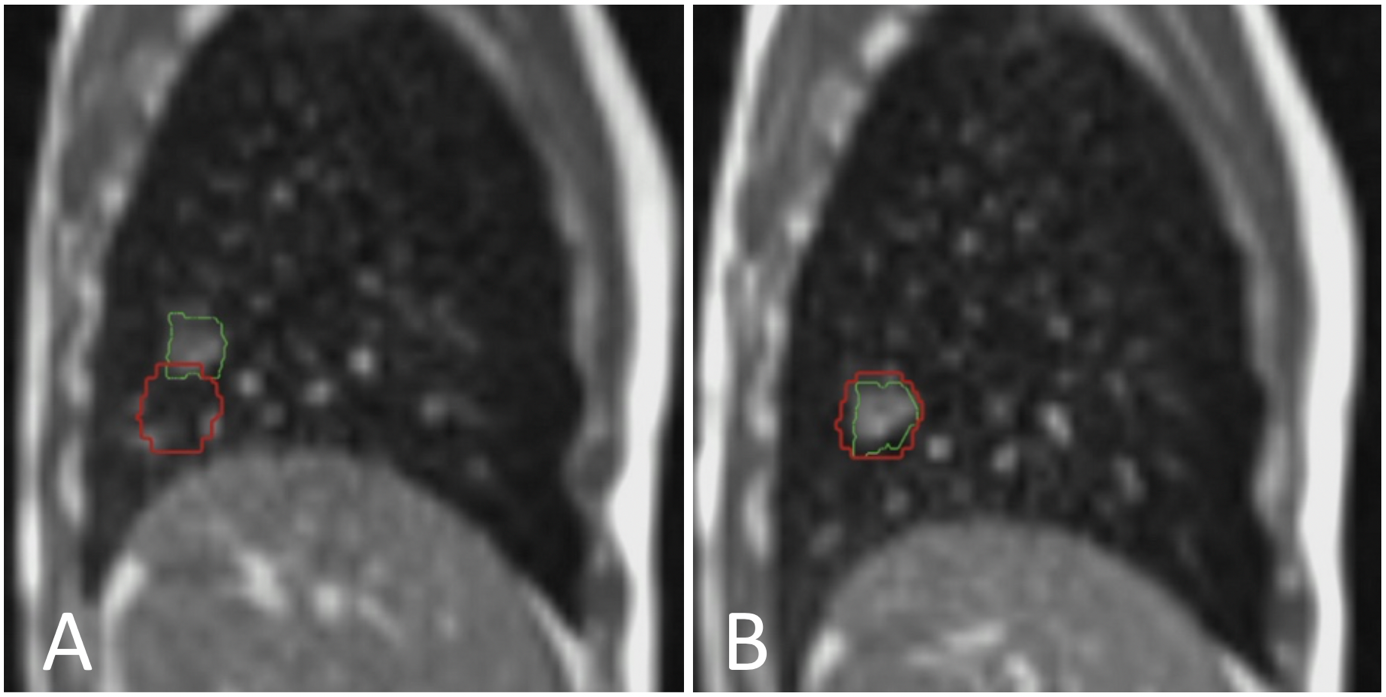


Supplementary Figure 1: Single-fraction lung stereotactic ablative radiotherapy using magnetic resonance (MR)-guidance is delivered in repeated breath-holds, with visual feedback provided to the patient using an adjustable mirror directed at an in-room monitor. Continuous MR-tracking of the gross tumor volume (GTV_t_; green) is performed in sagittal plane using deformable image registration (A, B). During delivery, the beam is automatically turned off when a specified proportion of the GTV_t_ is outside the gating window boundary (red).

Supplementary Table 1: Characteristics of patients treated with single-fraction lung stereotactic ablative radiotherapy (1 x 34 Gy) using magnetic resonance-guidance.

| **Case** | **Sex** | **Age** | **ECOG PS** | **Diagnosis** | **Biopsy** | **Location** | **GTV (cc)** | **PTV (cc)** |
| --- | --- | --- | --- | --- | --- | --- | --- | --- |
| 1 | Female | 75 | 2 | Lung cancer | No (second tumor, after a previous proven NSCLC) | RLL | 2.3 | 9.2 |
| 2 | Male | 75 | 0 | Metastasis (HNC) | Yes | RUL | 3.4 | 13.2 |
| 3 | Female | 71 | 1 | Lung cancer | No | RLL | 3.5 | 13.9 |
| 4 | Male | 80 | 2 | Metastasis (RCC) | No | LUL | 4.3 | 9.4 |
| 5 | Male | 76 | 0 | Lung cancer (NSCLC) | Yes | LLL | 3.3 | 11.7 |
| 6 | Male | 79 | 1 | Lung cancer | No | RLL | 2.5 | 10.4 |
| 7 | Male | 65 | 1 | Lung cancer | No (second tumor, after a previous proven NSCLC) | RUL | 1.8 | 7.5 |
| 8 | Female | 67 | 1 | Lung cancer | No | LLL | 2.5 | 9.8 |
| 9 | Female | 60 | 1 | Lung cancer | No | RLL | 6.5 | 20.5 |
| 10 | Male | 58 | 1 | Lung cancer | No | LLL | 1.9 | 8.4 |

*Abbreviations: ECOG PS, Eastern Cooperative Oncology Group Performance Status; GTV, gross tumor volume; PTV, planning target volume; HNC, head and neck cancer; RCC, renal cell carcinoma; NSCLC, non-small cell lung cancer; RLL, right lower lobe; RUL, right upper lobe; LLL, left lower lobe; LUL, left upper lobe.*
